# The insulator protein CTCF regulates *Drosophila* steroidogenesis

**DOI:** 10.1242/bio.012344

**Published:** 2015-05-15

**Authors:** Ujué Fresán, Sergi Cuartero, Michael B. O'Connor, M. Lluisa Espinàs

**Affiliations:** 1 Institute of Molecular Biology of Barcelona, IBMB-CSIC, and Institute for Research in Biomedicine IRB, Barcelona 08028, Spain; 2 Department of Genetics, Cell Biology and Development, University of Minnesota, Minneapolis, MN 55455, USA

**Keywords:** CTCF, Ecdysone, Halloween genes, Developmental timing

## Abstract

The steroid hormone ecdysone is a central regulator of insect development. In this report we show that CTCF expression in the prothoracic gland is required for full transcriptional activation of the Halloween genes *spookier*, *shadow* and *noppera-bo*, which encode ecdysone biosynthetic enzymes, and for proper timing of ecdysone-responsive gene expression. Loss of CTCF results in delayed and less synchronized larval development that can only be rescued by feeding larvae with both, the steroid hormone 20-hydroxyecdysone and cholesterol. Moreover, CTCF-knockdown in prothoracic gland cells leads to increased lipid accumulation. In conclusion, the insulator protein CTCF is required for Halloween gene expression and cholesterol homeostasis in ecdysone-producing cells controlling steroidogenesis.

## INTRODUCTION

The proper development of multicellular organisms requires accurate timing of specific developmental programs of gene expression. These timed programs involve signaling systems that respond to nutritional and environmental cues to direct coordinated developmental responses throughout the animal, such as transitions in morphology. In insects, these transitions include molting and metamorphosis which occur at regularly defined intervals and depend on pulses of the steroid hormone ecdysone (Yamanaka et al., 2013). During larval and pupal stages of insect development, the prothoracic gland (PG) is the tissue responsible for the synthesis of ecdysone that is secreted and converted into the active steroid molting hormone 20-hydroxyecdysone (20E) in target tissues, where it induces the activation of target genes. The neuropeptide protoracicotropic hormone (PTTH) is produced by two pairs of lateral neurosecretory cells in the insect brain and is released to signal to the PG (McBrayer et al., 2007). PTTH, through activation of its receptor torso and the mitogen-activated protein kinase (MAPK) cascade (Rewitz et al., 2009), leads to increased transcription of ecdysone biosynthetic enzymes encoded by the Halloween gene family (Gilbert, 2004). Transcriptional regulation of these genes is still poorly understood, although some transcription factors and chromatin modifiers have recently been identified for some of the biosynthetic genes (Danielsen et al., 2014; Deng and Kerppola, 2013; Moeller et al., 2013; Pankotai et al., 2010; Parvy et al., 2005). Halloween genes trigger the production of ecdysone from dietary cholesterol or phytosterols, since arthropods are unable to synthesize cholesterol. In recent years, evidence has accumulated that other factors in addition to PTTH, especially nutritional signals, act on the PG to control ecdysteroidogenesis (Layalle et al., 2008).

The insulator protein CTCF is a highly conserved zinc finger protein that has been shown to play an essential role in regulation of chromatin organization and gene expression during development both, in Drosophila and mammals (Herold et al., 2012). Recent data supports a role for insulator proteins in the organization of developmentally regulated intra- and interchromosomal contacts that are responsible for the diverse functions of CTCF in gene regulation, including context-dependent transcriptional activation, repression, insulation and imprinting (Phillips and Corces, 2009).

In this paper we show that CTCF is required for Halloween gene expression and cholesterol homeostasis in the prothoracic gland controlling steroidogenesis in Drosophila.

## RESULTS AND DISCUSSION

### CTCF controls the timing of larval development

**Fig. 1.**
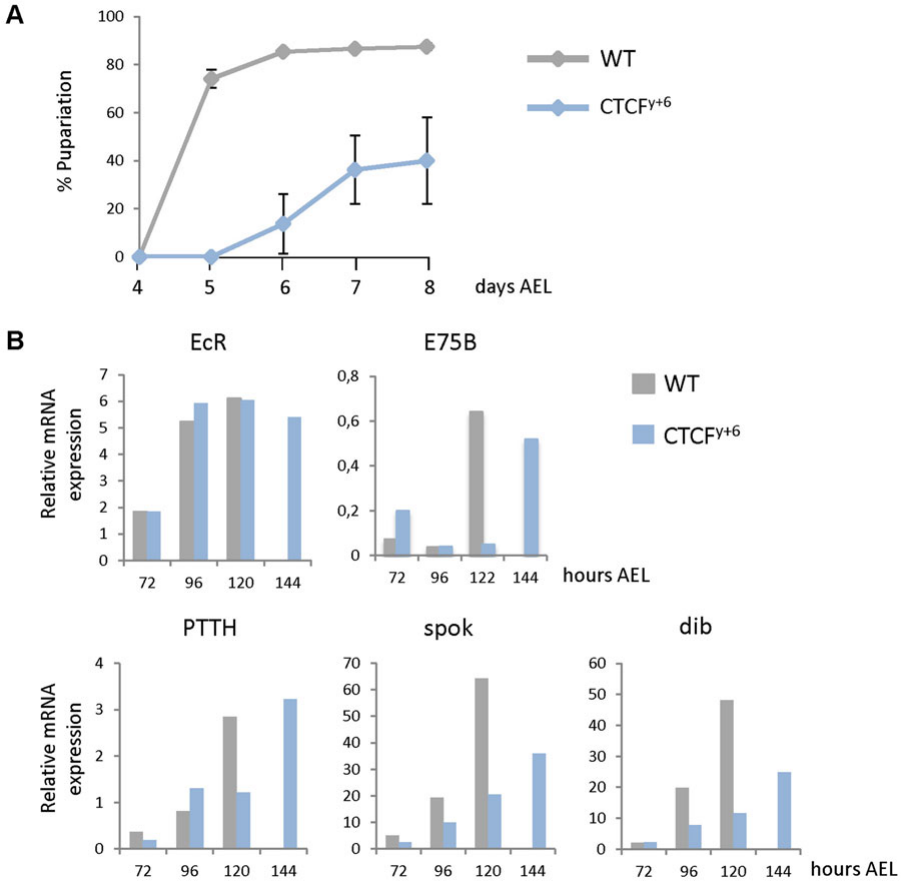
**Loss of CTCF produces developmental delay.** (A) The percentages of larvae of the indicated genotypes that underwent pupariation were plotted relative to the time in days after egg laying. Error bars show s.d. of three independent experiments. (B) Representative transcriptional profiles along time in hours after egg laying of the indicated genes in wild type and CTCF^y+6^ mutant larvae.

The analysis of Drosophila developmental timing in *CTCF^y+6^* null mutant background (Gerasimova et al., 2007) revealed that larval development was prolonged by two days on average in those mutant animals that progressed to the larval/pupal transition (Fig. 1A). We confirmed that the developmental delay in these animals was due to the loss of CTCF by performing rescue experiments using the UAS/Gal4 system. It has been reported that expression of *UAS-CTCF* with ubiquitous drivers lead to embryonic lethality while a single heat-shock during larval development of animals containing a *hsp70-Gal4* transgene (*hs>*) was sufficient to phenotypically rescue the majority of CTCF mutant flies (Mohan et al., 2007). Our analyses showed that a short pulse of CTCF significantly rescued the developmental delay in *CTCF^y+6^* mutants (supplementary material Fig. S1). Indeed, although 90% of *CTCF^y+6^* larvae died before pupariation after a 20 min heat-shock, some larvae did reach the pupal stage but pupariation was delayed by more than 3 days. CTCF expression was able to partially rescue both, lethality (around 75% of mutant larvae pupariate, data not shown) and, importantly, developmental delay (pupariation was clearly advanced by CTCF expression in the *CTCF^y+6^* mutant background, supplementary material Fig. S1).

The delayed pupariation of *CTCF^y+6^* mutants suggested that the ecdysone signaling pathway might be affected in these animals. Since the insulator protein CTCF plays an essential role in regulation of gene expression, we analyzed the transcript levels of components acting at different steps in ecdysone signaling pathway. It has been previously reported that the ecdysone-induced protein 75B locus contains a CTCF site involved in recruitment of the insulator protein CP190 in response to the hormone and proper activation of gene expression (Wood et al., 2011). Thus, we analyzed the expression of the ecdysone-responsive gene E75B during larval development. We also analyzed the transcript levels of the Ecdysone receptor (EcR), which is activated by hormone to regulate expression of ecdysone-responsive genes, PTTH, which is the peptide hormone that initiates steroid production, and the Halloween genes *spookier* (*spok*) (Ono et al., 2006) and *disembodied* (*dib*) (Chavez et al., 2000), two ecdysone biosynthetic enzymes expressed in the PG. Our analyses showed no changes in *EcR* transcriptional levels in CTCF mutant animals and only a delay in the induction of the expression of *E75B* and *PTTH*, each of which eventually showed transcript levels similar to controls (Fig. 1B). However, we observed a reduction in the transcriptional induction of two genes involved in ecdysone biosynthesis, *spok* and *dib*. These results suggest that CTCF might regulate induction of certain Halloween genes necessary for ecdysone synthesis.

### Loss of CTCF in ecdysone-producing cells impairs Halloween gene transcriptional activation

**Fig. 2.**
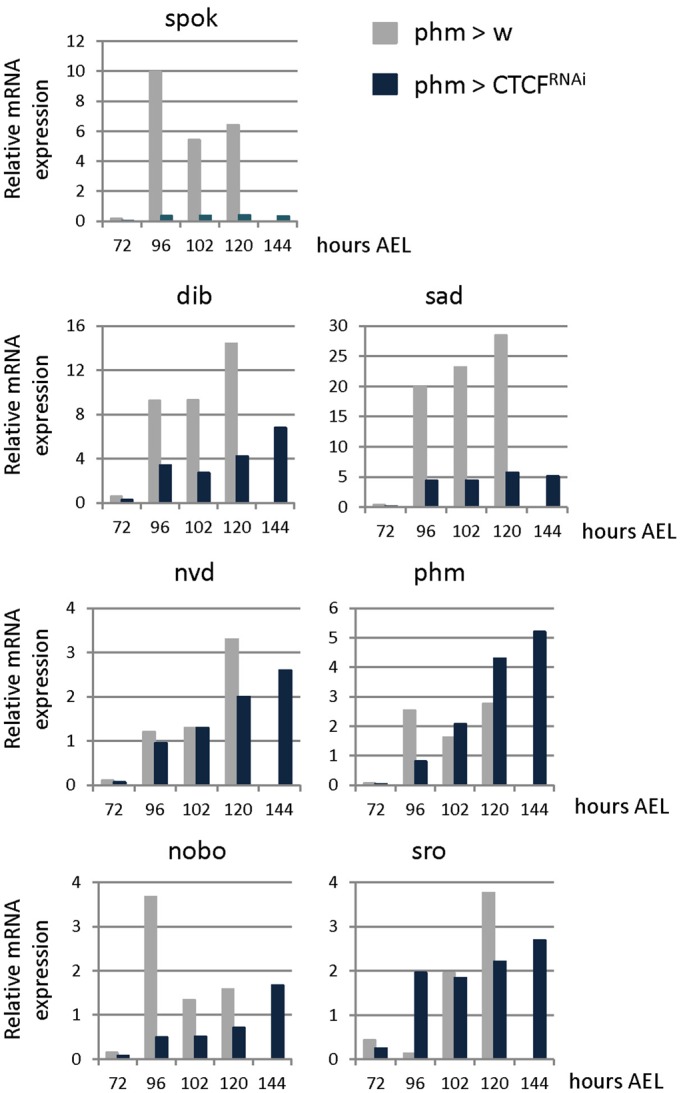
**CTCF knockdown in the prothoracic gland regulates expression of the Halloween genes.** Representative transcriptional profiles verses time in hours after egg laying of the Halloween genes *spok*, *dib*, *sad, nvd, phm, nobo* and *sro* in control (phm>w) and CTCF RNAi (phm>CTCF^RNAi^) larvae.

In order to analyze whether CTCF is required autonomously in the ecdysone-producing cells themselves to regulate Halloween gene expression we knocked down CTCF specifically in the PG using the UAS/Gal4 system. A *UAS-CTCF-RNAi* construct combined with *UAS-dicer2* was expressed in the prothoracic cells using a PG-specific *phantom-Gal4* (*phm>*) driver. We then measured the transcript levels of the Halloween genes *spok*, *dib*, *shadow* (*sad*) (Warren et al., 2002), *neverland* (*nvd*) (Yoshiyama et al., 2006), *phantom* (*phm*) (Niwa et al., 2004; Warren et al., 2004), *noppera-bo* (*nobo*) (Enya et al., 2014) and *shroud* (*sro*) (Niwa et al., 2010). Control larvae that only express *phantom-Gal4* in a wild type background exhibit a clear expression increase of these genes during larval development (Fig. 2). The expression of these Halloween genes is initially quite low during the early third-instar stage and then rises, as previously reported (McBrayer et al., 2007). In *phm>CTCF^RNAi^* background the transcript levels of *dib*, *sad* and *nobo* were 2-5 fold lower in *phm>CTCF^RNAi^* larvae at the time points of maximal induction (Fig. 2) and we observed almost no induction of *spok*, which is one of the genes of the so-called Black-Box that has been suggested to be a rate limiting step in ecdysone synthesis (Warren et al., 2009). On the other hand, no clear differences were observed for the Halloween genes *nvd*, *phm* and *sro* indicating that CTCF is required specifically in ecdysone-producing cells for activation of several, but not all of the ecdysone biosynthetic enzymes.

We investigated the possibility that loss of CTCF in the PG might indirectly affect ecdysone production by modifying PTTH signaling. First we analyzed whether the gene is expressed normally and found that *PTTH* transcripts are only found in the PG neurons in each brain hemisphere as in controls and the transcript levels were not decreased. In fact they were actually increased, during the prolonged larval stage (supplementary material Fig. S2A and B). We also analyzed the expression of the receptor *torso* and found similar transcript levels in *phm>CTCF^RNAi^* knockdown as in control larvae (supplementary material Fig. S2C). Therefore, our data indicate that loss of CTCF in the PG does not likely interfere with PTTH signaling to the PG.

### CTCF expression in the PG is required for proper timing of ecdysone-signaling gene expression

**Fig. 3.**
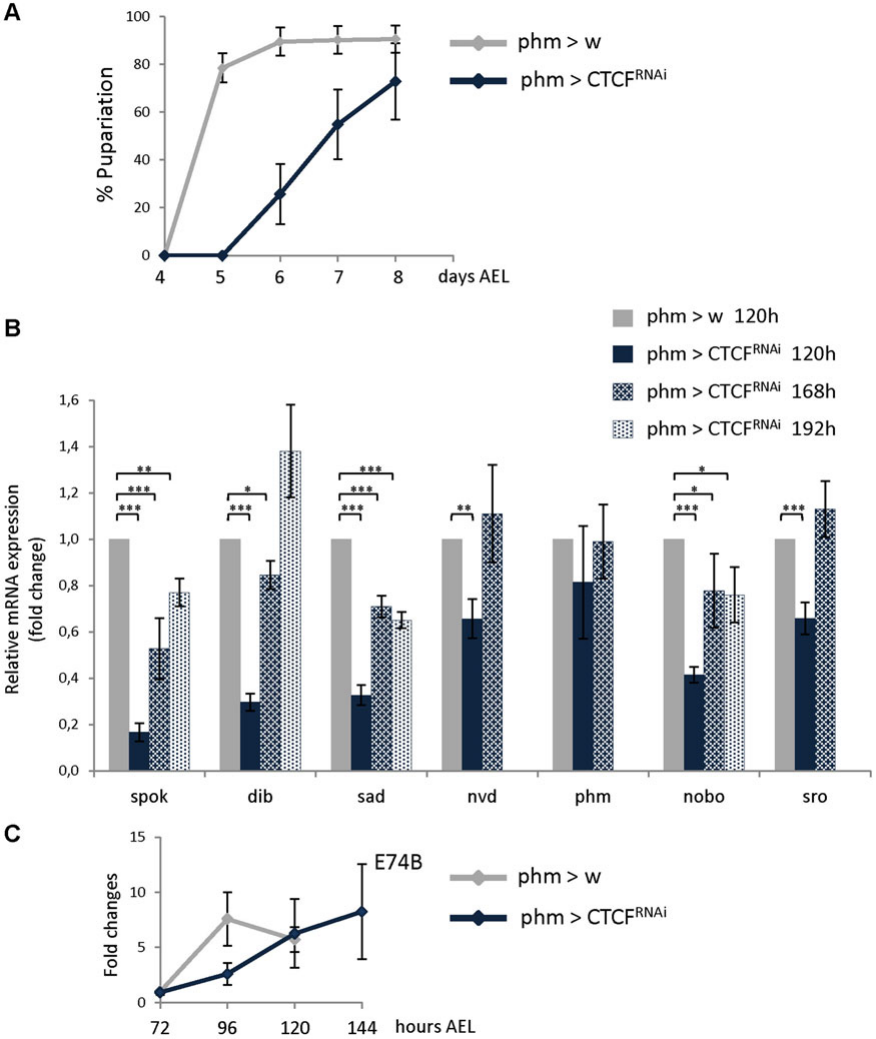
**The increase in ecdysone signaling is delayed in CTCF knockdown larvae.** (A) The percentages of larvae of the control (phm>w) and CTCF RNAi (phm>CTCF^RNAi^) genotypes that underwent pupariation were plotted relative to the time in days after egg laying. Error bars show s.d. of three independent experiments. (B) Transcriptional levels of Halloween genes in CTCF RNAi at the end of larval development as fold changes relative to control (phm>w 120 h). Error bars show s.e.m. of at least three independent experiments. ***P<0.001, **P<0.01, *P<0.1 with Student's *t*-test. (C) Temporal profile of ecdysone signaling gene *E74B* transcriptional induction in control and CTCF RNAi larvae. Error bars show s.e.m. of three independent experiments.

Reduced levels of CTCF in the PG led to partial lethality at different larval stages, but many knockdown animals were able to pupariate although most died before eclosion. We analyzed developmental timing in *phm>CTCF^RNAi^* knockdown larvae and we found that larval development was prolonged by two days on average and was less synchronous than control animals (Fig. 3A). We also measured the average time to ecdysis from the second to the third instar stage and found that it was increased by around 8 h for CTCF knockdown animals (supplementary material Fig. S3).

Since larval development was prolonged by two-three days in *phm>CTCF^RNAi^* knockdown animals, we analyzed Halloween gene expression at 168 and 192 h AEL. We found that transcript levels of *dib*, *nvd*, *phm* and *sro* increased as in control animals during the prolonged larval development (Fig. 3B). In contrast, even though at the end of larval development there was some increase in *spok*, *sad* and *nobo* transcription, transcript levels of these genes were always significantly lower in *phm>CTCF^RNAi^* compared to control animals (Fig. 3B). Moreover, difference for *spok* and *nobo* gene expression between control and CTCF knockdown animals is even higher if one considers that in control larva maximum expression is seen at 96 h and not 120 h of larval development (Fig. 2).

Interestingly, despite low transcriptional levels of some ecdysone biosynthetic genes in *phm>CTCF^RNAi^* animals during larval development, some animals are able to eventually mount a sufficient hormone response to initiate metamorphosis. To analyze whether there is a perturbation of ecdysone accumulation in these animals, we measured the transcript levels of the ecdysone-responsive gene *E74B* as a proxy for the ecdysone titer during the larval stage. In control larvae transcript levels of *E74B* showed a peak at 96 h AEL while in CTCF knockdown larvae a similar peak was reached but two days later (Fig. 3C), consistent with the length of the developmental delay exhibited by these mutant animals. These results are similar to that seen in animals lacking PTTH which mount a proper, but asynchronous, transcriptional response to ecdysone (McBrayer et al., 2007). Taken together our results indicate that the production of ecdysone, not the response to the hormone, is the source of delay in CTCF knockdown animals.

We also analyzed the participation of the insulator proteins CP190 and Ibf1/2 whose binding sites were previously shown to significantly overlap with CTCF-binding sites (Cuartero et al., 2014). We found that developmental timing was normal in both *phm>CP190^RNAi^* and *phm>Ibf1/2^RNAi^* larvae (data not shown), suggesting that the CP190-Ibf1/2 insulator complex does not participate in CTCF-mediated regulation of Drosophila steroidogenesis.

### Developmental timing depends on CTCF-mediated cholesterol homeostasis and ecdysone synthesis

**Fig. 4.**
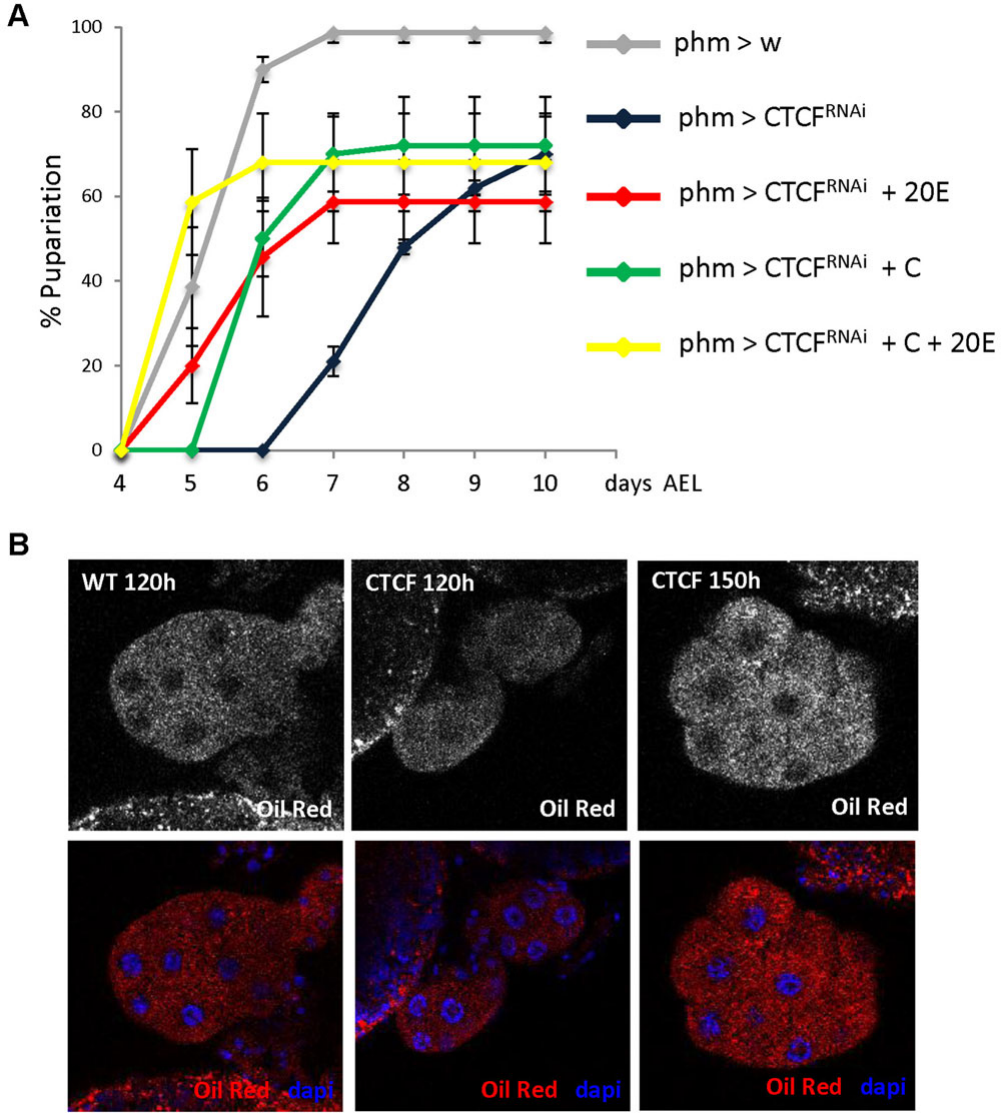
**Both, 20-hydroxyecdysone (20E) and cholesterol (C) are required to rescue CTCF knockdown phenotypes.** (A) Control (phm>w) and CTCF RNAi (phm>CTCF^RNAi^) larvae were fed food containing EtOH, 20E or/and C and the percentages of larvae of the indicated genotypes that underwent pupariation were plotted relative to the time in days after egg laying. Error bars show s.d. of three independent experiments. (B) Lipid accumulation in control and CTCF RNAi ecdysone-producing cells. Single plane confocal micrographs showing lipid droplets in PG cells stained with Oil Red O (either in black and white, upper panels, or in red, lower panels) of 120 h AEL clear-gut *phm>w* control larvae (left panels), 120 h AEL blue-gut *phm>CTCF^RNAi^* larvae (middle panels) and 150 h AEL clear-gut *phm>CTCF^RNAi^* larvae (right panels). DNA was labeled with DAPI (blue).

Though *phm>CTCF^RNAi^* knockdown larvae showed low transcriptional levels of the Halloween genes *spok*, *sad* and *nobo*, they are able to induce ecdysone-responsive genes such as *E74B* and most larvae still progressed to the larval/pupal transition. However, pupariation was delayed in these animals, suggesting that CTCF is needed for proper timing of high level ecdysone production. To determine if low levels of ecdysteroids are responsible for the developmental delay, we fed either 20-hydroxyecdysone (20E), which is the active form of the hormone, or cholesterol (C), which is the precursor for ecdysone biosynthesis in the PG, to *phm>CTCF^RNAi^*. Although some of the 20E-fed CTCF-knockdown larvae started to pupariate at the same time as control animals, individuals of the *phm>CTCF^RNAi^* population pupariate over a longer period of time (Fig. 4A). Since it has been recently reported that Ecdysone (E) and 20E have different effects on developmental timing of wild type larvae (Ono, 2014), we also fed E to *phm>CTCF^RNAi^* larvae but we did not observe any differences with respect to 20E-fed larvae (data not shown). On the other hand, when larvae were cholesterol-fed puparium formation and adult eclosion were normal although delayed by an average of one day. Indeed, we were only able to efficiently rescue developmental delay and adult eclosion by feeding larvae with both, 20E and C, suggesting that CTCF is needed not only to synthesize ecdysone from cholesterol but also to mediate cholesterol homeostasis in PG cells.

It has been reported that at the onset of pupariation there is an increase of lipid droplets in PG cells that can be seen by Oil Red O staining of precisely staged 120 h AEL wandering larvae (Talamillo et al., 2013). At 120 h AEL, *phm>CTCF^RNAi^* PGs had a reduced content of lipid droplets in comparison to controls (Fig. 4B, compare middle and left panels), likely due to developmental delay. However, there is also an increase of lipid droplets at the end of larval development in *phm>CTCF^RNAi^* PG cells which tend to be slightly higher than in control animals (Fig. 4B, compare right and left panels and supplementary material Fig. S4). Since the subcellular lipid accumulation phenotype of *phm>CTCF^RNAi^* PG cells is similar to that of Niemann-Pick type C (*npc*) mutants (Huang et al., 2005), we analyzed *dnpc1a* transcriptional levels but we found no changes between control and *phm>CTCF^RNAi^* larvae (data not shown). Increased lipid accumulation in the fat body in EcR knockdown larvae has been reported (Kamoshida et al., 2012), but our results show that *EcR* transcriptional levels do not change in CTCF mutant animals (Fig. 1B). On the other hand, as shown above, CTCF expression in the prothoracic gland is required for transcriptional activation of the Halloween gene *nobo* (Fig. 2), which has been shown to be necessary for normal accumulation of cholesterol in PG cells (Enya et al., 2014). Thus, the abnormal accumulation of lipids droplets in CTCF knockdown animals are in agreement with CTCF-mediated regulation of *nobo* gene expression. These data along with results reported above showing partial rescue in cholesterol-fed CTCF-knockdown larvae (Fig. 4A) suggest a role of CTCF in sterol homeostasis in the PG.

In recent years evidence has accumulated that PTTH is a major, but not the only, developmental signal that triggers ecdysteroidogenesis; multiple factors, in particular nutritional signals reflecting the general metabolic status of the larvae, act directly on the PG to control ecdysteroidogenesis. Insulin and Target of Rapamycin (TOR) pathways have been shown to play a direct role in regulating ecdysone synthesis in the PG (Caldwell et al., 2005; Colombani et al., 2005; Layalle et al., 2008; Mirth et al., 2005). Insulin signaling has been studied extensively in Drosophila as a regulator of tissue growth and involves activation of several downstream factors including PI3K and AKT. In contrast, PTTH signals through activation of the receptor Torso and the Erk pathway (Rewitz et al., 2009). Interestingly, it has been reported that CTCF plays a role in insulin-induced cell proliferation and acts downstream of insulin-induced activation of both Erk and Akt (Gao et al., 2007; Tsui et al., 2014). In addition to insulin/TOR, recent work has shown that TGFβ/activin signaling is also essential for ecdysone synthesis and developmental transitions (Gibbens et al., 2011). It has been suggested that *dSmad2*, the major downstream mediator of TGFβ signals, might participate directly in regulating Halloween gene transcription. The observation that TGFβ promotes complexes between Smad proteins and CTCF on the H19 imprinting control region (Bergstrom et al., 2010), raises the possibility that these complexes could also regulate Halloween gene transcription. We searched for CTCF-binding sites in the promoter regions of Halloween genes and we found motifs at less than −1 kb from TSSs in all of them except *nvd* (supplementary material Fig. S5). Nevertheless, it remains to be determined whether CTCF-mediated induction of these genes is mediated through these sites and whether complexes with other transcription factors might also be involved.

### Conclusions

Here we show that the insulator protein CTCF plays important roles in Drosophila steroidogenesis at two different levels: on one hand, CTCF controls cholesterol homeostasis in the PG and, on the other hand, CTCF is required for activation of the enzymes responsible for the synthesis of the hormone. Whether these two events are coupled needs further analyses but our data suggest that this is unlikely since to efficiently rescue of developmental delay in CTCF-knockdown larvae required both, ecdysone and cholesterol. Overall, our results suggest that the insulator protein CTCF might be part of a molecular mechanism that couple nutritional input and timing of juvenile maturation.

## MATERIALS AND METHODS

### Fly stocks

All flies were raised at 25°C on standard medium. The following transgenic and mutant flies were used: *CTCF^y+6^* (Gerasimova et al., 2007), *phm-Gal4* (Rewitz et al., 2009), *hsp70-Gal4* (Bloomington Drosophila Stock Center #2077), *UAS-dCTCF* (Mohan et al., 2007) *UAS-CTCF-RNAi* (Vienna Drosophila RNAi Center-VDRC #30713), *UAS-CP190-RNAi* (VDRC #35078), *UAS-Ibf1-RNAi* (VDRC #35426), *UAS-Ibf2-RNAi* (VDRC #42121) and *UAS-dicer2* (VDRC #60008).

### Staging larvae, developmental timing analyses and feeding experiments

Females were allowed to lay eggs on apple juice agar plates for 2 h. Synchronized new hatched first-instar larvae were placed on standard medium supplemented with yeast at 25°C and allowed to develop to the desired time points. For developmental timing analyses, 30 larvae were placed per vial and pupariation timing was scored periodically. A single heat shock (37°C) for 20 min at 24 h of development was performed in rescue experiments. Feeding experiments were performed by adding 20-Hydroxyecdysone (Santa Cruz) at 0.33 mg/ml in ethanol and/or Cholesterol (Sigma) at 0.35 mg/ml. Control larvae were fed with ethanol.

### Quantitative RT-PCR

Total RNAs were extracted from ring gland+brain of precisely staged larvae with TRIzol Reagent (Life Technologies) and purified with RNeasy Mini Kit (Qiagen). cDNAs were prepared from 0.5 µg of RNA using the Transcriptor First Strand cDNA Synthesis Kit (Roche). qRT-PCR was performed on Roche LightCycler 480 System (Roche) using LightCycler 480 SYBER Green I Master (Roche). Three independent biological replicates for each genotype at each time point were performed. Transcriptional levels were normalized to ribosomal protein RpL23. Primers used are listed in supplementary material Table S1.

### Oil Red O staining

Ring gland+brain were fixed in 4% paraformaldehyde for 20 min, washed twice in PBS and stained with Oil Red O (Sigma) solution at 0.06% in isopropanol for 30 min. Samples were washed and stained with DAPI before mounting in Vectashield (Vector Laboratories). Single plane images were taken with a confocal Leica TCS SP2-AOBS microscope.

## Supplementary Material

Supplementary Material

## References

[BIO012344C1] Bergstrom R., Savary K., Moren A., Guibert S., Heldin C.-H., Ohlsson R. and Moustakas A. (2010). Transforming growth factor beta promotes complexes between Smad proteins and the CCCTC-binding factor on the H19 imprinting control region chromatin. *J. Biol. Chem.* 285, 19727-19737. 10.1074/jbc.M109.088385 20427289 PMC2888383

[BIO012344C2] Caldwell P. E., Walkiewicz M. and Stern M. (2005). Ras activity in the Drosophila prothoracic gland regulates body size and developmental rate via ecdysone release. *Curr. Biol.* 15, 1785-1795. 10.1016/j.cub.2005.09.011 16182526

[BIO012344C3] Chavez V. M., Marques G., Delbecque J. P., Kobayashi K., Hollingsworth M., Burr J., Natzle J. E. and O'Connor M. B. (2000). The Drosophila disembodied gene controls late embryonic morphogenesis and codes for a cytochrome P450 enzyme that regulates embryonic ecdysone levels. *Development* 127, 4115-4126. 10976044 10.1242/dev.127.19.4115

[BIO012344C4] Colombani J., Bianchini L., Layalle S., Pondeville E., Dauphin-Villemant C., Antoniewski C., Carré C., Noselli S. and Léopold P. (2005). Antagonistic actions of ecdysone and insulins determine final size in Drosophila. *Science* 310, 667-670. 10.1126/science.1119432 16179433

[BIO012344C5] Cuartero S., Fresan U., Reina O., Planet E. and Espinas M. L. (2014). Ibf1 and Ibf2 are novel CP190-interacting proteins required for insulator function. *EMBO J.* 33, 637-647. 10.1002/embj.201386001 24502977 PMC3989656

[BIO012344C6] Danielsen E. T., Moeller M. E., Dorry E., Komura-Kawa T., Fujimoto Y., Troelsen J. T., Herder R., O'Connor M. B., Niwa R. and Rewitz K. F. (2014). Transcriptional control of steroid biosynthesis genes in the Drosophila prothoracic gland by ventral veins lacking and knirps. *PLoS Genet.* 10, e1004343 10.1371/journal.pgen.1004343 24945799 PMC4063667

[BIO012344C7] Deng H. and Kerppola T. K. (2013). Regulation of Drosophila metamorphosis by xenobiotic response regulators. *PLoS Genet.* 9, e1003263 10.1371/journal.pgen.1003263 23408904 PMC3567155

[BIO012344C8] Enya S., Ameku T., Igarashi F., Iga M., Kataoka H., Shinoda T. and Niwa R. (2014). A Halloween gene noppera-bo encodes a glutathione S-transferase essential for ecdysteroid biosynthesis via regulating the behaviour of cholesterol in Drosophila. *Sci. Rep.* 4, 6586 10.1038/srep06586 25300303 PMC4192634

[BIO012344C9] Gao J., Li T. and Lu L. (2007). Functional role of CCCTC binding factor in insulin-stimulated cell proliferation. *Cell Prolif.* 40, 795-808. 10.1111/j.1365-2184.2007.00472.x 18021171 PMC6496666

[BIO012344C10] Gerasimova T. I., Lei E. P., Bushey A. M. and Corces V. G. (2007). Coordinated control of dCTCF and gypsy chromatin insulators in Drosophila. *Mol. Cell* 28, 761-772. 10.1016/j.molcel.2007.09.024 18082602 PMC2579779

[BIO012344C11] Gibbens Y. Y., Warren J. T., Gilbert L. I. and O'Connor M. B. (2011). Neuroendocrine regulation of Drosophila metamorphosis requires TGFβ/Activin signaling. *Development* 138, 2693-2703. 10.1242/dev.063412 21613324 PMC3109597

[BIO012344C12] Gilbert L. I. (2004). Halloween genes encode P450 enzymes that mediate steroid hormone biosynthesis in Drosophila melanogaster. *Mol. Cell. Endocrinol.* 215, 1-10. 10.1016/j.mce.2003.11.003 15026169

[BIO012344C13] Herold M., Bartkuhn M. and Renkawitz R. (2012). CTCF: insights into insulator function during development. *Development* 139, 1045-1057. 10.1242/dev.065268 22354838

[BIO012344C14] Huang X., Suyama K., Buchanan J., Zhu A. J. and Scott M. P. (2005). A Drosophila model of the Niemann-Pick type C lysosome storage disease: dnpc1a is required for molting and sterol homeostasis. *Development* 132, 5115-5124. 10.1242/dev.02079 16221727

[BIO012344C15] Kamoshida Y., Fujiyama-Nakamura S., Kimura S., Suzuki E., Lim J., Shiozaki-Sato Y., Kato S. and Takeyama K.-i. (2012). Ecdysone receptor (EcR) suppresses lipid accumulation in the Drosophila fat body via transcription control. *Biochem. Biophys. Res. Commun.* 421, 203-207. 10.1016/j.bbrc.2012.03.135 22503687

[BIO012344C16] Layalle S., Arquier N. and Léopold P. (2008). The TOR pathway couples nutrition and developmental timing in Drosophila. *Dev. Cell* 15, 568-577. 10.1016/j.devcel.2008.08.003 18854141

[BIO012344C17] McBrayer Z., Ono H., Shimell M., Parvy J.-P., Beckstead R. B., Warren J. T., Thummel C. S., Dauphin-Villemant C., Gilbert L. I. and O'Connor M. B. (2007). Prothoracicotropic hormone regulates developmental timing and body size in Drosophila. *Dev. Cell* 13, 857-871. 10.1016/j.devcel.2007.11.003 18061567 PMC2359579

[BIO012344C18] Mirth C., Truman J. W. and Riddiford L. M. (2005). The role of the prothoracic gland in determining critical weight for metamorphosis in Drosophila melanogaster. *Curr. Biol.* 15, 1796-1807. 10.1016/j.cub.2005.09.017 16182527

[BIO012344C19] Moeller M. E., Danielsen E. T., Herder R., O'Connor M. B. and Rewitz K. F. (2013). Dynamic feedback circuits function as a switch for shaping a maturation-inducing steroid pulse in Drosophila. *Development* 140, 4730-4739. 10.1242/dev.099739 24173800 PMC3833430

[BIO012344C20] Mohan M., Bartkuhn M., Herold M., Philippen A., Heinl N., Bardenhagen I., Leers J., White R. A. H., Renkawitz-Pohl R., Saumweber H. et al. (2007). The Drosophila insulator proteins CTCF and CP190 link enhancer blocking to body patterning. *EMBO J.* 26, 4203-4214. 10.1038/sj.emboj.7601851 17805343 PMC2230845

[BIO012344C21] Niwa R., Matsuda T., Yoshiyama T., Namiki T., Mita K., Fujimoto Y. and Kataoka H. (2004). CYP306A1, a cytochrome P450 enzyme, is essential for ecdysteroid biosynthesis in the prothoracic glands of Bombyx and Drosophila. *J. Biol. Chem.* 279, 35942-35949. 10.1074/jbc.M404514200 15197185

[BIO012344C22] Niwa R., Namiki T., Ito K., Shimada-Niwa Y., Kiuchi M., Kawaoka S., Kayukawa T., Banno Y., Fujimoto Y., Shigenobu S. et al. (2010). Non-molting glossy/shroud encodes a short-chain dehydrogenase/reductase that functions in the ‘Black Box’ of the ecdysteroid biosynthesis pathway. *Development* 137, 1991-1999. 10.1242/dev.045641 20501590

[BIO012344C23] Ono H. (2014). Ecdysone differentially regulates metamorphic timing relative to 20-hydroxyecdysone by antagonizing juvenile hormone in Drosophila melanogaster. *Dev. Biol.* 391, 32-42. 10.1016/j.ydbio.2014.04.004 24727669

[BIO012344C24] Ono H., Rewitz K. F., Shinoda T., Itoyama K., Petryk A., Rybczynski R., Jarcho M., Warren J. T., Marqués G., Shimell M. J. et al. (2006). Spook and Spookier code for stage-specific components of the ecdysone biosynthetic pathway in Diptera. *Dev. Biol.* 298, 555-570. 10.1016/j.ydbio.2006.07.023 16949568

[BIO012344C25] Pankotai T., Popescu C., Martin D., Grau B., Zsindely N., Bodai L., Tora L., Ferrus A. and Boros I. (2010). Genes of the ecdysone biosynthesis pathway are regulated by the dATAC histone acetyltransferase complex in Drosophila. *Mol. Cell. Biol.* 30, 4254-4266. 10.1128/MCB.00142-10 20584983 PMC2937542

[BIO012344C26] Parvy J.-P., Blais C., Bernard F., Warren J. T., Petryk A., Gilbert L. I., O'Connor M. B. and Dauphin-Villemant C. (2005). A role for betaFTZ-F1 in regulating ecdysteroid titers during post-embryonic development in Drosophila melanogaster. *Dev. Biol.* 282, 84-94. 10.1016/j.ydbio.2005.02.028 15936331

[BIO012344C27] Phillips J. E. and Corces V. G. (2009). CTCF: master weaver of the genome. *Cell* 137, 1194-1211. 10.1016/j.cell.2009.06.001 19563753 PMC3040116

[BIO012344C28] Rewitz K. F., Yamanaka N., Gilbert L. I. and O'Connor M. B. (2009). The insect neuropeptide PTTH activates receptor tyrosine kinase torso to initiate metamorphosis. *Science* 326, 1403-1405. 10.1126/science.1176450 19965758

[BIO012344C29] Talamillo A., Herboso L., Pirone L., Pérez C., González M., Sánchez J., Mayor U., Lopitz-Otsoa F., Rodriguez M. S., Sutherland J. D. et al. (2013). Scavenger receptors mediate the role of SUMO and Ftz-f1 in Drosophila steroidogenesis. *PLoS Genet.* 9, e1003473 10.1371/journal.pgen.1003473 23637637 PMC3630131

[BIO012344C30] Tsui S., Dai W. and Lu L. (2014). CCCTC-binding factor mediates effects of glucose on beta cell survival. *Cell Prolif.* 47, 28-37. 10.1111/cpr.12085 24354619 PMC3946918

[BIO012344C31] Warren J. T., Petryk A., Marques G., Jarcho M., Parvy J.-P., Dauphin-Villemant C., O'Connor M. B. and Gilbert L. I. (2002). Molecular and biochemical characterization of two P450 enzymes in the ecdysteroidogenic pathway of Drosophila melanogaster. *Proc. Natl. Acad. Sci. USA* 99, 11043-11048. 10.1073/pnas.162375799 12177427 PMC123207

[BIO012344C32] Warren J. T., Petryk A., Marqués G., Parvy J.-P., Shinoda T., Itoyama K., Kobayashi J., Jarcho M., Li Y., O'Connor M. B. et al. (2004). Phantom encodes the 25-hydroxylase of Drosophila melanogaster and Bombyx mori: a P450 enzyme critical in ecdysone biosynthesis. *Insect Biochem. Mol. Biol.* 34, 991-1010. 10.1016/j.ibmb.2004.06.009 15350618

[BIO012344C33] Warren J. T., O'Connor M. B. and Gilbert L. I. (2009). Studies on the Black Box: incorporation of 3-oxo-7-dehydrocholesterol into ecdysteroids by Drosophila melanogaster and Manduca sexta. *Insect Biochem. Mol. Biol.* 39, 677-687. 10.1016/j.ibmb.2009.08.004 19699302

[BIO012344C34] Wood A. M., Van Bortle K., Ramos E., Takenaka N., Rohrbaugh M., Jones B. C., Jones K. C. and Corces V. G. (2011). Regulation of chromatin organization and inducible gene expression by a Drosophila insulator. *Mol. Cell* 44, 29-38. 10.1016/j.molcel.2011.07.035 21981916 PMC3190163

[BIO012344C35] Yamanaka N., Rewitz K. F. and O'Connor M. B. (2013). Ecdysone control of developmental transitions: lessons from Drosophila research. *Annu. Rev. Entomol.* 58, 497-516. 10.1146/annurev-ento-120811-153608 23072462 PMC4060523

[BIO012344C36] Yoshiyama T., Namiki T., Mita K., Kataoka H. and Niwa R. (2006). Neverland is an evolutionally conserved Rieske-domain protein that is essential for ecdysone synthesis and insect growth. *Development* 133, 2565-2574. 10.1242/dev.02428 16763204

