# Supplementary material for: The insulator protein CTCF regulates Drosophila steroidogenesis
Source: Biol Open. 2015 May 15;4(7):852–7. doi: 10.1242/bio.012344 (PMC4571099; doi:10.1242/bio.012344)
Supplement: Supplementary Material [file supp_4_7_852__index.html]

The insulator protein CTCF regulates Drosophila steroidogenesis — The insulator protein CTCF regulates Drosophila steroidogenesis — Supplementary Material 

# The insulator protein CTCF regulates *Drosophila* steroidogenesis

## BIO012344 Supplementary Material

- Supplementary Material
