# Supplementary material for: The insulator protein CTCF regulates Drosophila steroidogenesis
Source: Biol Open. 2015 May 15;4(7):852–7. doi: 10.1242/bio.012344 (PMC4571099; doi:10.1242/bio.012344)
Supplement: Supplementary Material [file supp_bio.012344_BIO012344supp.pdf]

Supplementary material:

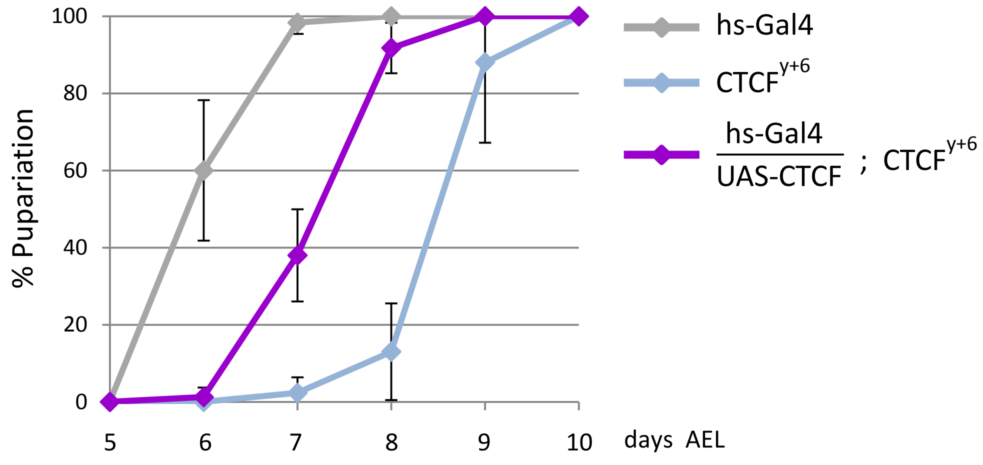

Fig. S1. Rescue of homozygous *CTCF*$^{y+6}$ developmental delay by expression of CTCF using *hsp70-Gal4* driver and a single heat shock (37ºC) for 20 min at 24 hours of development. The percentages of larvae of the indicated genotypes from total larvae that underwent pupariation were plotted relative to the time in days after egg laying. The increased delay observed in control and *CTCF*$^{y+6}$ compared to results shown in Fig. 1A are due to the heat shock treatment.

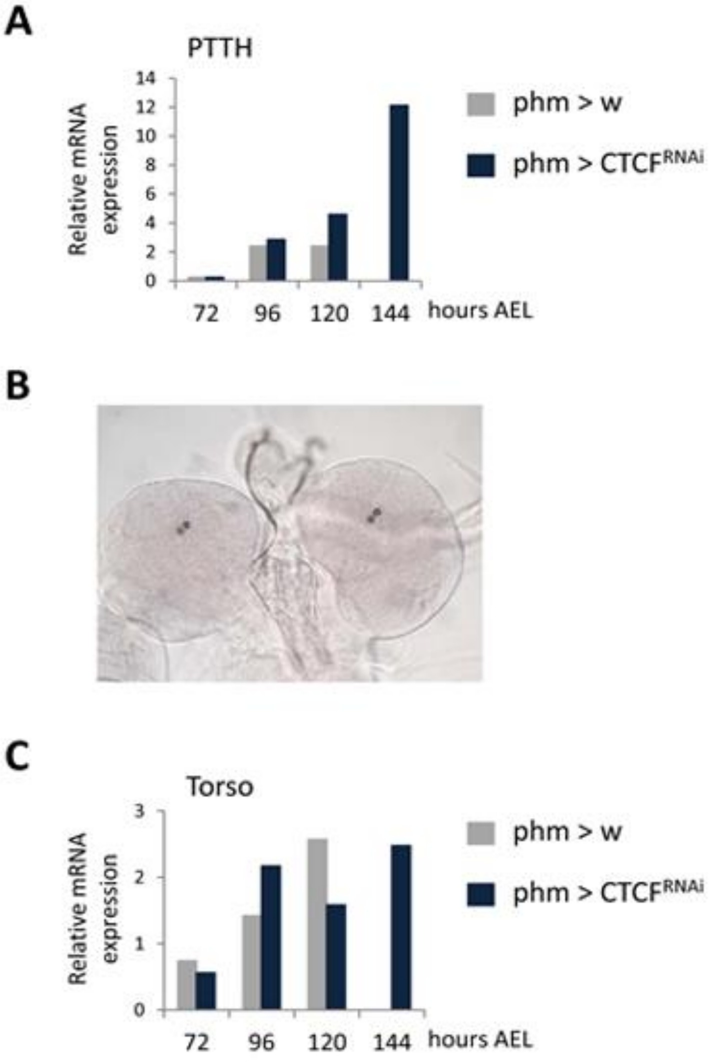

Fig. S2. (A) Representative transcriptional profile along time in hours after egg laying of *PTTH* in control and CTCF RNAi larvae. Transcriptional levels were measured by RT-qPCR and normalized to ribosomal protein RpL23. (B) In situ hybridization shows *PTTH* expression on *phm>CTCF*^{RNAi} larval brain. (C) Representative transcriptional profile along time in hours after egg laying of *torso* in control and CTCF RNAi larvae. Transcriptional levels were measured by RT-qPCR and normalized to ribosomal protein RpL23.

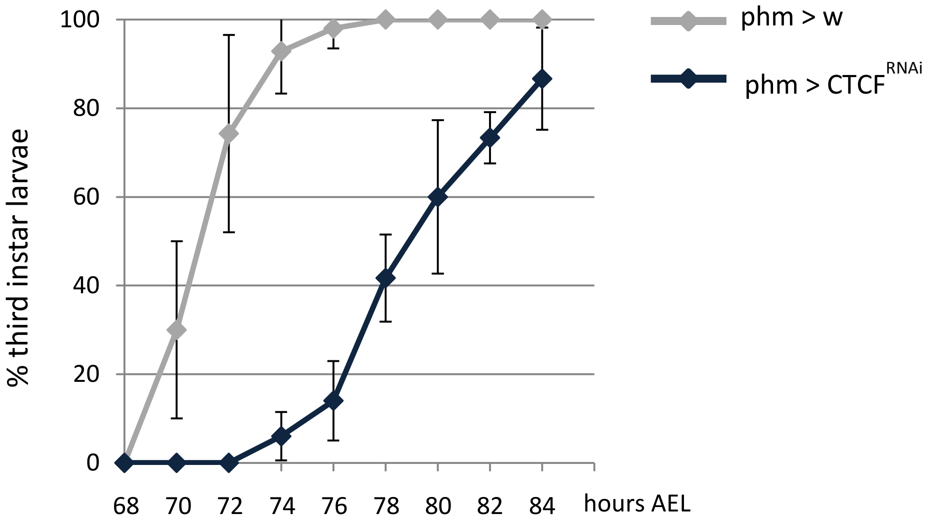

Fig. S3. The percentages of larvae of the indicated genotypes that had undergone 2nd to 3rd instar transition were plotted relative to the time in hours after egg laying. Error bars show SD of at least three independent experiments.

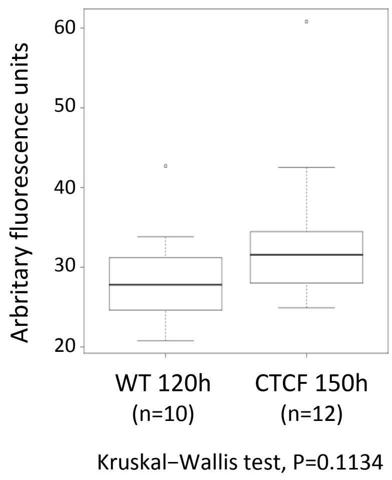

Fig. S4. Box plot graph showing quantification of fluorescence intensity in Oil Red O stained PG cells.

A

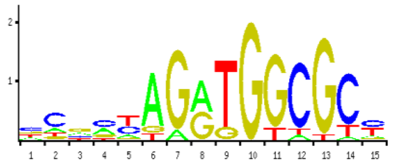

B

Gene	Motif sequence	Distance from TSS	Genomic position
dib	tgaattgatgttgcc	−363	3L:4061915..4061929
nobo	ctgccaagtggatca	−689	2R:13239040..13239026
nvd	-		
phm	tccacagatcgcgcg	−965	X:18578820..18578834
sad	gggatggggggagca	−316	3R:7702627..7702641
sro	caactaggtgtttct<br>tgagaaaatggctct<br>aacaataatggcgtt	−184<br>−243<br>−863	3R:29794413..29794399<br>3R:29794340..29794354<br>3R:29793720..29793734
spok	ctttaagatggtgcc<br>cagacagatggatac	−107<br>−280	3RHet:2158896..2158910<br>3RHet:2159055..2159069

Figure S5. (A) The logo representation of CTCF consensus motif. (B) CTCF motifs found in genomic regions between −1kb and TSS of the Halloween genes using the JASPAR CORE database and default settings.

Table S1. Primers used for qRT-PCR experiments.

Gene	Forward	Reverse
dib	TGCCCTCAATCCCTATCTGGTC	ACAGGGTCTTCACACCCATCTC
E74B	CGCGAGTTCAAAGTGCTCTA	GGAGGGAGAGTGGTGGTGT
E75B	GTGGTGGGATGAAGCACGTA	GAAGTGGTGGTCAGTAGGGC
EcR (all isoforms)	TGCGAAGAAGAGCAAGAAGG	CAGGTGAGGGCGTTGTAGTG
nobo	TCAAGGGCGAGCAATTCCAA	ACAAACTACCGCCCTCATCG
nvd	GGAAGCGTTGCTGACGACTGTG	TAAAGCCGTCCACTTCCTGCGA
phm	GGATTTCTTTCGGCGCGATGTG	TGCCTCAGTATCGAAAAGCCGT
PTTH	GTTTGGACGAGATGGTGGGT	TTCGATGTACTGCGATCGGG
RpL23	GACAACACCGGAGCCAAGAACC	GTTTGCGCTGCCGAATAACCAC
sad	CCGCATTCAGCAGTCAGTGG	ACCTGCCGTGTACAAGGAGAG
spok	TATCTCTTGGGCACACTCGCTG	GCCGAGCTAAATTTCTCCGCTT
sro	AGCAGCTGAAGGTCGATAGC	TCCGACTTGATGTTGTGGCA
Torso	CCAGTCAGAGCGATGTTTGGT	GACGGATATGGCATTCCACC
